# Validity and test retest reliability of the vascular quality of life Questionnaire-6: a short form of a disease-specific health-related quality of life instrument for patients with peripheral arterial disease

**DOI:** 10.1186/s12955-017-0762-1

**Published:** 2017-09-29

**Authors:** Christine Kumlien, Joakim Nordanstig, Mats Lundström, Monica Pettersson

**Affiliations:** 1 The Department of Care Science, Malmö University, Jan Waldenströms gata 25, 20506 Malmö, Sweden; 2 The Department of Cardio-Thoracic and Vascular Surgery, Skane University Hospital, Malmö, Sweden; 3 The Department of Vascular Surgery and Institute of Medicine, Department of Molecular and Clinical Medicine, Sahlgrenska University Hospital and Academy, Gothenburg, Sweden; 4 Department of Clinical Sciences, Ophthalmology, Faculty of Medicine, Lund University, Lund, Sweden; 5 The Institute of Health and Care Science, the Department of Vascular Surgery, Sahlgrenska University Hospital and Academy, Gothenburg, Sweden

**Keywords:** Peripheral arterial disease, Health-related quality of life, VascuQoL, VascuQoL-6, Cognitive interviews, Rasch analysis

## Abstract

**Background:**

Many existing patient-reported outcome measures are extensive regarding both patient burden and administration, and in terms of analysing and reporting results. The VascuQoL-6 (VQ6) – a short version of the original Vascular Quality of Life Questionnaire (VascuQoL), a disease-specific instrument for peripheral arterial disease – was recently developed. However, the VQ6 has not yet been empirical tested with regard to content validity, construct validity and test retest reliability. Our aim was, therefore, to explore both the validity and the reliability of the VQ-6 in a target population with established peripheral arterial disease.

**Methods:**

Two hundred patients treated at two vascular centres were consecutively recruited for the survey. Administered questionnaires included VQ6 and the Short Form Health Survey-36 (SF-36). Out of the 200 patients, 150 also received a second VQ6 questionnaire for a test-retest assessment. Further, a purposive sample of 22 patients consented to participate in cognitive interviews. All included patients suffer from peripheral arterial disease. The questionnaire data was tested by both Rasch analysis and traditional psychometric methods, while the cognitive interviews were analysed descriptively.

**Results:**

The validity and reliability of the VQ6, as tested in a target population without the surrounding 19 items from the original VascuQoL, was high, in general, and a good fit to the Rasch model was observed. Further, an excellent internal consistency and significant correlations between comparable dimensions in SF-36 were demonstrated. In the test-retest analysis, the percentage agreement was somewhat poor (<70%) in the six items. However, no systematic disagreements between the two assessments were seen in any of the six items, and the test-retest assessment for the VQ6 sum score showed an acceptable intraclass correlation coefficient (0.86). Finally, all items in the VQ6 were considered as both understandable and relevant by the interviewed patients.

**Conclusions:**

The VQ6 has acceptable to good psychometric properties with regard to data quality, scale assumptions, targeting, validity and reliability. Further, VQ6 seems to be easy to use and comprehend within the target population of patients with PAD.

## Background

During the last decades, health-related quality of life (HRQoL) has become an increasingly important measure within the health care systems when evaluating outcome and quality of care [1]. This is especially important for patients with chronic disorders such as peripheral arterial disease (PAD) where a complete cure of the underlying atherosclerotic disease is not possible. The main goal of vascular surgical treatment is to provide relief of symptoms and to improve functional ability and quality of life. Further goals are to prevent progression of the systemic atherosclerotic disease and to mitigate the risk for cardiovascular events and limb loss [2–4]. It is well established that the HRQoL is significantly impaired in patients suffering from PAD [5–7]. Symptoms of PAD range from intermittent claudication – characterised by exercise-induced leg pain, cramps or aching, − to critical limb ischaemia that often manifests as ischemic rest pain and/or non-healing ulcers or gangrene [2]. The PAD symptoms are associated with limitations in physical functions and difficulties in performing daily life activities [8]. Therefore, outcomes that address patients’ own perceptions about how PAD affects their daily life are important. However, many of the existing patient-reported outcome measures are too extensive for practical clinical use regarding the following: patient burden, health-care staff administration and scoring, and in terms of analysing and reporting results.

There are several generic and disease-specific health-related quality of life measurements available for use in patients with PAD. Short Form 36 Health Survey (SF-36) [9] and EuroQol 5 dimensions (EQ5D) [10] are two common valid and reliable generic instruments that have been frequently used to evaluate the PAD disease burden [5, 6] and outcome following invasive vascular procedures [7, 11, 12]. Of these two instruments, EQ5D is less extensive, including only five items. However, generic instruments are limited by their broad approach, which may reduce both sensitivity and responsiveness to clinical change when evaluating a certain treatment in a specific patient group. Disease-specific instruments focus on dimensions of function considered the most important for the target group of interest. Vascular Quality of Life Questionnaire (VascuQoL) is a PAD-specific instrument [13] recommended as one of the preferred questionnaires when evaluating HRQoL outcomes in patients with PAD [14–18]. Recently, a short form of the original VascuQol questionnaire was developed: VascuQol-6 (VQ6) [19]. The VQ6 is a six-item questionnaire, including the most efficient items from the original instrument that demonstrated strong correlations between scores from the original VascuQol and the short version. VQ6 was developed using a combination of qualitative and quantitative methodology. Rasch analysis was used for item reduction [20], preserving the most informative items and removing redundant items. The selected items were thereafter confirmed as relevant from the patient’s perspective by cognitive interviews [19]. However, the VQ6 was developed based on a theoretic model (the Rasch model), with all items from VascuQoL included as a starting point; therefore, further knowledge about the validity and the reliability of VQ6 without the surrounding items from the original VascuQoL is needed. Moreover, the VQ6 has not yet been empirically tested with regard to content validity, construct validity and test retest reliability. Our aim with this study was, therefore, to explore the validity and the reliability of the VQ-6 in a target population with established PAD.

## Methods

This was a psychometric study including both quantitative and qualitative methods in a vascular surgical setting at two university hospitals in southern Sweden.

### Study population

Patients with peripheral arterial disease (*n* = 219) treated at the two vascular centres were consecutively invited to the study; in total, 200 patients (91%) accepted participation. Inclusion criteria were a recent invasive vascular procedure and the ability to understand and answer a questionnaire in Swedish. Further, a purposive sample of 22 patients diagnosed with PAD and treated at either of the vascular centres were invited during their in-hospital stay to participate in cognitive interviews, to which they all consented. Inclusion criteria were that they had undergone an invasive vascular treatment for PAD, had the ability to speak and understand Swedish and could take part in an interview.

### Data collection

All data collection was performed during 2014. A vascular nurse invited the patients to participate in the study when they attended the one-month follow-up visit after an invasive vascular procedure. They were asked to fill out the questionnaires during the same outpatient visit. Out of the 200 patients, 150 patients also received a second questionnaire for a test-retest assessment, together with a prepaid envelope, to be answered at home one week after their visit at the out-patient clinic and the first questionnaire response. Demographic data, baseline risk factors and comorbidity were collected from the medical records.

Three nursing students conducted the cognitive interviews during the patients’ in-hospital stay. As a gatekeeper, the head nurse selected the patients in accordance with the inclusion criteria and invited them to participate. The interviews concerned how understandable and relevant each item was perceived and were dichotomously rated by yes or no. Further, the patients were asked to describe the meaning of each item in their own words and to indicate if there were any words or phrases that were difficult to comprehend. This procedure was performed to test whether the respondents comprehended the questions in the intended way in order to strengthen the content validity [21, 22].

Both verbal and written information about the study was given, and the participants provided written informed consent. The study was approved by the Regional Ethical Review Board at Lund University (no 315/2008 and no 750/2013).

### Questionnaires

#### VascuQoL- 6

The VascuQoL-6 (VQ6) is a short form (including six items) of the original VascuQoL (including 25 items) questionnaire and aims to reflect the entire spectrum of PAD, i.e., both intermittent claudication and critical ischemia (19). The intention with VQ6 is to measure the latent trait regarding the influence of PAD in daily life by including items that reflect the five dimensions of the original VascuQoL. The six items concern limitation in activities (activity), tiredness in the legs (symptom), walking ability (activity), concerns about poor circulation in the legs (emotional aspects), ability to take part in social activities (social aspects) and discomforts from pain in the leg (pain). Each question has a four-point response scale (1 most problems - 4 no problems) (Table 1). A sum raw score can be calculated, ranging from 6 to 24, by summarizing the score on each question. A higher value indicates a better health status. Through Rasch analysis, an average person Rasch score can be calculated. The VQ-6 has shown excellent psychometric properties in a previous study based on a theoretical model [19].

**Table 1:** VascuQol 6 items

1 Because of the poor circulation in my legs, the range of activities that I would have liked to do in the past two weeks has been.... (1 severely limited-4 Not at all limited)
2. During the past two weeks, my legs felt tired or weak.... (1 All of the time-4 None of the time)
3 During the past two weeks, because of the poor circulation in my legs, my ability to walk has been.... (1 Extremely limited-4 Not at all limited)
4 During the past two weeks, I have been concerned about having poor circulation in my legs.... (1 All of the time-4 None of the time)
5 During the past two weeks, because of the poor circulation in my legs, my ability to participate in social activities has been.... (1 Totally limited- 4 Not at all limited)
6 During the past two weeks, when I have had pain in the leg (or foot) it has given me.... (1 A great deal of discomfort or distress-4 No discomfort or distress)

#### Short form health Survey-36

Short Form Health Survey-36 (SF-36) is a generic well-established health-related quality of life instrument designed to assess generic health concepts that are not specific to age, disease or treatment group [9]. It consists of 36 items in eight health domains: bodily pain, physical functioning, role limitations due to physical problems, mental health, vitality, social functioning, role limitations due to emotional problems and general health. The response rate ranges from yes or no to a three-to-six response scale. Two summary components can be calculated: physical component score (PSC) and mental component score (MCS) [23]. All domain score results are converted to a scale ranging from 0 (worst possible score) to 100 (best possible score), while PCS and MCS scores are transformed so that a score of 50 represents the general Swedish population mean. The standard version of the questionnaire with a four-week recall period was used [9].

### Psychometric and statistical methods

#### Descriptive statistics and correlation analysis

Baseline demographic data and risk factors are presented as mean ± standard deviation (SD) and absolute and relative frequencies. The non-parametric Mann-Whitney test were used to compare the VQ6 scores in patients who underwent open surgery versus endovascular treatment as well as theVQ6 scores in patients with different PAD severity (i.e. intermittent claudication versus critical limb ischemia). A *p*-value at <0.05 was considered as significant. The correlation between the eight domains, PCS and MCS in the SF-36 and the six items in the VQ6 were analysed using Spearman’s correlation coefficient, reflecting the latent construct of VQ6, i.e., construct validity. Finally, internal consistency was analysed by Cronbach’s alpha. Descriptive and correlation analysis were performed using SPSS version 22.0 (SPSS Inc., Chicago, IL, USA).

#### Rasch analysis

Rasch analysis defines what is required from item response data to describe capability or personalities [24]. The model separately locates persons and items on the same linear logit (log-odd units) metric, which can take values from minus to plus infinity (with mean item location set at zero). Data that accord sufficiently with the model enable invariant linear measurement and comparisons [24, 25].

The response scale was tested by exploring the three category thresholds between the four response options in category probability curves. The category thresholds must be ordered and well separated from each other indicating that the respondents chose a response option in a predicted way and not by random. This means that patients should be able to distinguish adequately between neighboring response categories; for example being able to distinguish in item 2 between “some of the time” and “a little of the time”. Item fit statistics means exploring if each item contributes to the respondent’s capability in a predictable way. This test produces two measures: infit and outfit mean square. Both measures should have a value of 1.0. An infit mean square > 1 indicates more variation in the observed data than predicted by the model. An outfit mean square < 1 indicates less variation in the observed response pattern than predicted. Accepted limits are from 0.7 to 1.3.

When exploring how many levels of person-ability that the questionnaire can discriminate, i.e., measurement precision, the test results in two measures: person separation and a separation reliability coefficient. A person separation of 2.00 is the minimum accepted level of discrimination for an instrument to produce a valid measure. A lower person separation indicates that the instrument may not be sensitive enough to distinguish in this case, between high or low impact of the vascular disease.

The items in a questionnaire should preferably measure the same underlying trait and no other dimensions (so-called unidimensionality). This is analysed by standardized residual variance, and the raw variance explained by measures should be >60%. The instrument’s capacity in targeting was explored through an item map, which shows if the difficulty of the items and the ability of the respondents have about the same mean. There should be easy items for poorly performing patients and difficult items for the able patients. Finally, the Rasch analysis can explore if there exists differential item functioning (DIF). DIF means that a certain item behaves systematically different for different groups of patients. A large DIF may cause poor fitting of data to the Rasch model. In the present study, DIF was tested between groups of different age and sex. The Rasch analysis was performed using the WINSTEPS software, Version 3.92.0 (Winsteps, Beaverton, OR, USA).

#### Test-retest reliability

The test-retest on the item level was analysed using statistical software for evaluating paired ordered data [26]. This method allows calculation of possible variations between assessments at both individual and group level displayed by frequency distributions of every pair of assessments in a cross tabulation. Percentage agreement (PA) describes the proportion of unchanged assessments, and a PA exceeding 70% is considered as acceptable agreement [26]. Relative position (RP) demonstrates a systematic shift in the position on the scale between two assessments at a group level towards a lower or higher rate of disturbance on the second assessment. The relative concentration (RC) visualises a systematic variation in the concentration of the two assessments at group level. Possible values of RP and RC range between −1 and 1. The value 0 (zero) means no variations between assessments, and a statistical significant difference between assessments is considered when the 95% confidence interval (CI) does not include zero. A relative operating characteristic (ROC) curve obtained by plotting the pairs of cumulated proportions against each other with the zero point was used to illustrate the systematic level of change. A concave or convex curve is a sign of systematic disagreement in position, while an S-shaped curve will be displayed if the raters concentrate their assessments differently on the scale categories. Relative variation (RV) demonstrates the individual difference from the systematic pattern of changes between the two assessments. The RV value could be up to 1, and a 95% CI that does not cover zero indicates that individual variations must be accounted when interpreting the result. RV <0.1 is, in general, regarded as negligible [27]. Missing data were excluded pairwise.

The correlation between the VQ6 sum score at the first and second assessment was analysed by Spearman’s correlation test. Further, a two-way mixed intraclass correlation coefficient (ICC) was calculated, including a two-way ANOVA. An ICC exceeding 0.70 indicates excellent test-retest reliability [28], and a *p*-value of <0.05 was considered as significant. A Bland-Altman plot analysis was performed in order to evaluate potential bias between the mean differences in VQ6 sum scores and to estimate the 95% confidence interval of the difference between the two testing occasions [29].

#### Cognitive interviews

The number of patients that rated the six items in the VQ6 as understandable and relevant was summarized. Comments concerning the meaning and interpretation of the six items were analysed descriptively.

## Results

The mean age of the 200 patients who answered the questionnaire was 72 years; 111 patients (55%) were male. The majority were patients with critical limb ischemia (70%), with many having undergone endovascular treatment (67%). Ninety patients (44%) were current or previous smokers, and 111 (55%) suffered from hypertension (Table 2). There was no significant differences in any of the six VQ6 items,or the VQ6 sum score, in patients undergoing open surgery versus endovascular treatment or in patients with intermittent claudication versus critical limb ischemia.

**Table 2:** Demographics and risk factor characteristic in patient population, *N* = 200

	Total sample	Patients test retest *n* = 134
Age mean (SD)	72 (9.5)	71 (7.8)
Sex *n* (%)		
Male/female	111 (55)/89 (45)	71 (53)/63 (47)
Severity of disease *n* (%)		
Claudication	29 (15)	20 (15)
Critical ischemia	141 (70)	94 (70)
Other^a^	14 (7)	11 (8)
Missing	16 (8)	9 (7)
Type of intervention *n* (%)		
Open surgery	46 (23)	36 (27)
Endovascular	134 (67)	84 (63)
Combination open/endovascular	9 (4)	8 (6)
Missing	11 (6)	6 (4)
Risk factors *n* (%)		
Smoking current/previous	29 (14)/61(30)	20 (15)/42 (31)
Hypertension	111 (55)	36 (27)
Cardiac disease	57 (28)	75 (56)
Diabetes	60 (30)	36 (27)
Renal disease	25 (13)	28 (21)
Chronic pulmonary disease	33 (16)	15 (11)
Missing	24 (12)	16 (12)

^a^Patients classified as acute ischemia, graft occlusion or popliteal aneurysm

All eight dimensions, PCS and MCS in SF-36 were significantly correlated with all VQ6 items and the VQ6 sum score (*p*-value < 0.001). The strongest correlation was observed between PCS and VQ6 sum score (*r* = 0.69), symptom (item 2) (*r* = 0.64) activity and social (item 1and 5) (*r* = 0.63) (Table 3). The internal consistency for the VQ6 was 0.93.

**Table 3:** Cross-sectional construct validity, correlations between SF-36 domains and VQ6 items and VQ6 sum score

	Item 1 activity	Item 2 symptom	Item 3 activity	Item 4 emotional	Item 5 social	Item 6 pain	VQ6 sum score
Physical functioning	0.58	0.59	0.62	0.55	0.62	0.50	0.58
Role function physical	0.60	0.51	0.47	0.39	0.59	0.55	0.60
Bodily pain	0.45	0.52	0.49	0.36	0.49	0.59	0.45
General health	0.38	0.41	0.36	0.40	0.38	0.32	0.38
Vitality	0.47	0.51	0.43	0.37	0.44	0.43	0.47
Social functioning	0.53	0.44	0.43	0.35	0.56	0.43	0.53
Role function emotional	0.41	0.37	0.29	0.31	0.44	0.33	0.43
Mental health	0.31	0.36	0.29	0.47	0.38	0.32	0.39
PCS	0.63	0.64	0.63	0.51	0.63	0.58	0.69
MCS	0.33	0.29	0.20	0.27	0.37	0.26	0.34

Spearman’s rank correlation

All domains and VQ6 items were significantly correlated *p*-value < 0.001

*PCS* Physical component summary score, *MCS* Mental component summary score

### Rasch analysis

**Fig. 1:**
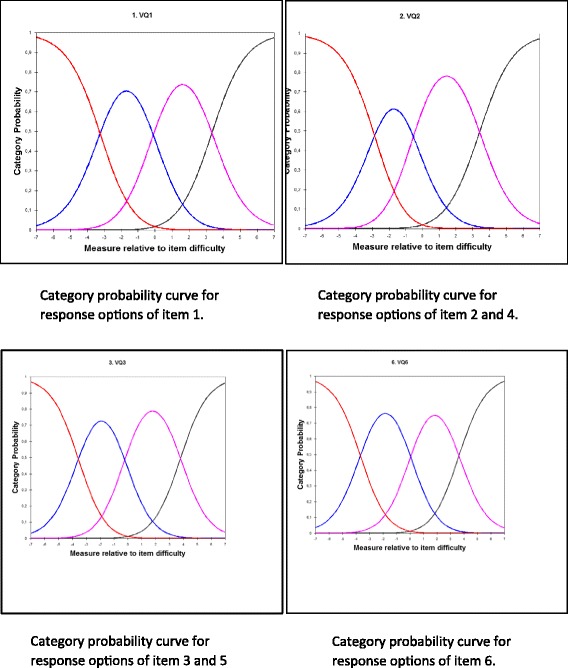
Category thresholds between the four response options in category probability curves for the six items in VQ6

The analysis of the category probability curves showed that all four response options were ordered (Fig. 1). Person separation was 3.03; and the person reliability coefficient was 0.90, indicating a high measurement precision. This means that the VQ6 is able to differentiate between at least three levels of vascular disease impact on patients HRQoL. Fit statistics showed no redundancy or outliers among the items. Infit mean square varied between 0.70 and 1.32, and outfit mean square varied between 0.70 and 1.32. Evidence of unidimensionality for VQ6 was demonstrated by residual analysis; the raw variance explained by measures was 72%, and the standardized residual variance of first contrast showed an eigenvalue of 1.52.

**Fig. 2:**
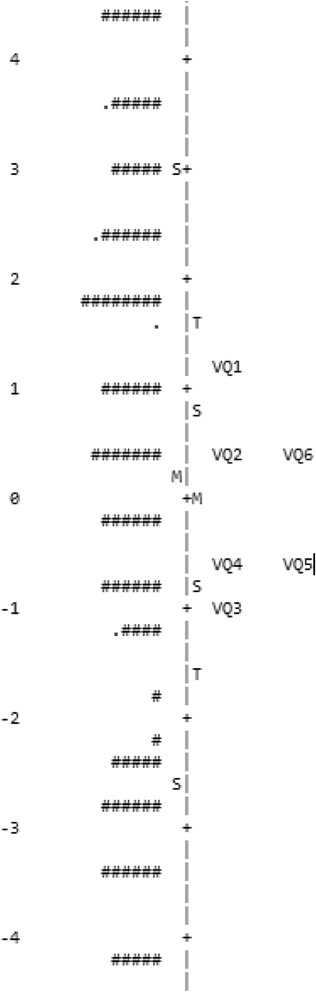
Person-item map for the six items in VQ6 showing the ability of the patients to the left and the difficulty of the items (VQ1-VQ6) to the right of a linear scale (from −6 to +6 logits). The measure (logit) shows the logarithm of the odds ratio for being able to perform an item activity successfully. The distance between a respondent and an item shows the probability of the respondents to perform successfully the item activity. Preferable, person and item should center on the same mean value. M, S and T represent mean, 1SD and 2SD, respectively. Each # is 2 persons. The instrument is well targeted because the item means and person means were separated by only 0.48 logits

The differential item functioning (DIF) showed a moderate DIF (0.82) for gender in item 4, but no DIF for age. Targeting was demonstrated by a Person mean of 0.48 logits, which means that the average person ability is a little higher than the average item difficulty (Fig. 2). Finally, the conclusion is that VQ6 fits the Rasch model very well.

### Test-retest

**Fig. 3:**
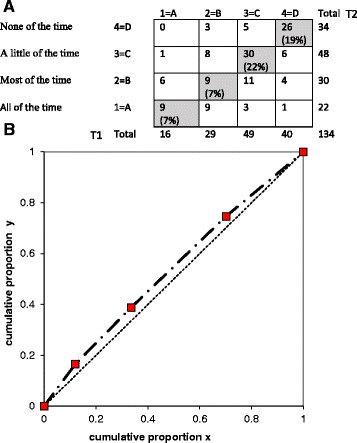
The contingency table (**a**) (T1 vertical = first measurement, T2 horizontal = second measurement) and the ROC curve (**b**) show no systematic disagree in concentration between the two assessments of item 4. ROC, relative operating characteristic

**Fig. 4:**
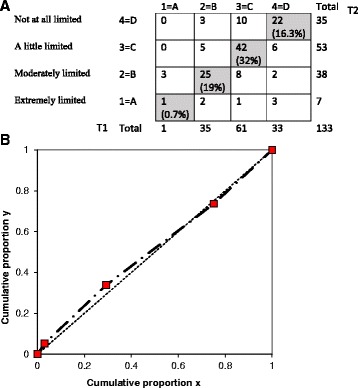
The contingency table (**a**) (T1 vertical = first measurement, T2 horizontal = second measurement) and the ROC curve (**b**) show no systematic disagree in concentration between the two assessments of item 3. ROC, relative operating characteristic

Out of the 150 patients who received a second questionnaire, 134 returned a response. The six items had percentage of agreement (PA), ranging from 55% (item 4) to 68% (item 3) (Table 4). The highest level of disagreement in relative position (RP) was for item 4 (−0.07), reflecting emotional aspects. The analysis of item 4 is displayed in Fig. 3 and the contingency table shows that 60 (45%) patients did not give the same response in both assessments. The ROC curve above the diagonal suggests that the patients more often used a lower alternative, representing more problem, in the second assessment. In comparison, the result for item 3 (activity) spreads both above and below the diagonal. The contingency table in Fig. 4 shows that 43 (32%) patients gave different responses at both assessments for item 3 and the slight S-shape of the ROC curve further illustrate this. The highest level of disagreement in (RC) was for item 3 (0.07). However, no significant systematic disagreement was seen either in RP or RC between the two assessments in any of the six items since all 95% confidence interval covered zero. The items with highest relative rank variance (RV), showing individual disagreements, were item 3 (0.13), item 1 (0.11) and item 6 (0.10), meaning that there were signs of individual variations.

**Table 4:** Test retest on item level

	PA %	RP (CI 95%)	RC (CI 95%)	RV (CI 95%)
Item 1 Activity	59	0.03 (−0.04–0.12)	−0.04 (−0.14–0.06)	0.11 (0.04–0.17)
Item 2 Symptom	60	−0.05 (−0.12–0.01)	0.02 (−0.07–0.11)	0.02 (0.01–0.04)
Item 3 Activity	68	−0.03 (−0.11–0.06)	0.07 (−0.15–0.01)	0.13 (0.04–0.22)
Item 4 Emotional	55	−0.07 (−0.15–0.00)	−0.02 (−0.12–0.08)	0.09 (0.03–0.14)
Item 5 Social	64	0.01 (−0.07–0.06)	0.03 (−0.05–0.12)	0.03 (0.01–0.05)
Item 6 Pain	63	−0.04 (−0.12–0.04)	−0.02 (−0.11–0.08)	0.10 (0.03–0.17)

*PA* = Percentage agreement, 100% means no change in the answers

*RP* = Relative position, *RC* = Relative concentration, RP and RC range from −1 to 1 and a systematic change occur when CI does not cover zero

*RV* = Relative rank variance, the higher RV the larger individual variation and zero means total absence of individual variance

**Fig. 5:**
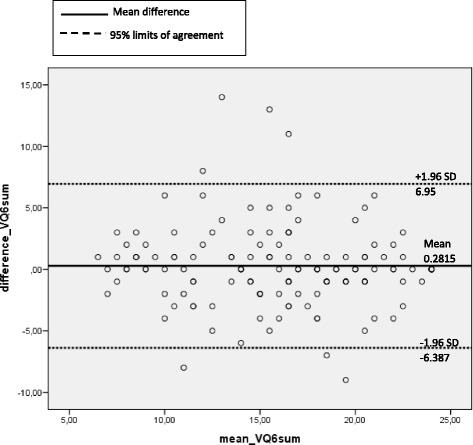
The Bland-Altman plot display the differences plotted against the averages of the two measurements of VQ6 sum score. The limits of agreement are defined as the mean difference ± 1.96SD of differences

The test-retest analysis for the VQ 6 sum score at the first and second assessment correlated significantly (*r* = 0.76); and average ICC was 0.86 (with a *p*-value of 0.31), indicating that there was no significant difference between the two assessments. In the Bland-Altman analysis, the mean VQ6 sum score did not exceed the 95% CI interval of limits of agreement indicating that the VQ6 sum scores were in agreement between the two measurements (Fig. 5).

### Cognitive interviews

**Fig. 6:**
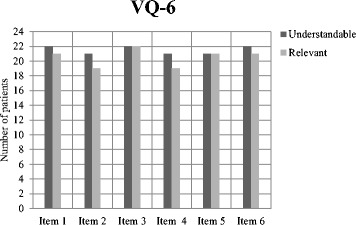
Cognitive interviews with patients (*n* = 22)

The majority of the patients answered that all items in the VQ6 were both understandable and relevant. Item 2 and 4 were perceived less relevant, and item 1, 3 and 6 were considered as understandable by all patients (Fig. 6).

The comments concerned the two-weeks recall period that each item in the VQ6 refers to and the meaning of some of the words. The time frame was considered as too short due to the chronic nature of the PAD.

*“Yes, in the first place I think that two weeks are completely unimportant to ask about. I mean that when you’ve had this for many years, another two weeks doesn’t make any difference. It’s getting worse and worse. It would have been better to ask about the last year. I think.”*

Words or phrases that were experienced as confusing were “been concerned of” (item 4), “activities” (item 1) and that “legs felt tired or weak” (item 2). The patients expressed feelings that were stronger and more complex than was captured in the specific items.

*“It may be that a stronger word than concerned is needed. I would rather express it like I am feeling anxious and irritated.”*

## Discussion

The validity and reliability of the VQ6 – tested in a target population without the surrounding 19 items from the original VascuQoL questionnaire – was, in general, high. Moreover, the VQ6 instrument was demonstrated to fit the Rasch model very well. Further, an excellent internal consistency and significant correlations between comparable dimensions in SF-36 were observed. In the test-retest analysis, the percentage agreement was somewhat poor in some of the six items. However, no significant systematic disagreement between the two assessments was observed in any of the six items. In addition, the test-retest for the VQ sum score showed an acceptable intra class correlation coefficient and did not exceed the limits of agreement in the Bland-Altman plot. Finally, the interviewed patients considered all items in  VQ6 to be both understandable and relevant.

Correlations between the dimensions of SF-36 and the items in the VQ6 reflecting comparable domains were somewhat stronger for the items representing physical and social activities in daily life than for the items representing symptoms and emotional aspects. Similar results were demonstrated in previous studies testing the original VascuQoL instrument [16, 17]. Items representing symptoms are often considered as causal indicators assuming to cause deterioration in HRQoL. Therefore, their impact may vary between patients. Causal indicators are more heterogeneous, which accordingly may result in a weaker correlation structure [30]. However, the present study showed significant correlations between all the eight dimensions, PCS and MCS in SF-36, the six items in VQ6 and VQ6 sum score, suggesting that the six items in VQ6 represent a high construct validity.

To obtain ordered thresholds according the Rasch analysis, it was necessary to reduce the response options from six in the original VascuQoL questionnaire to four in the VQ6 [19]. The present study confirmed the appropriateness of four response options; further, the response scale analysis showed that the category probability curves worked well and that the four response categories were ordered. The functioning of ordered rating scales response categories is crucial, as ordinal scales aim at illustrating complex phenomena even if the distance between response options is unknown [31]. This means that the response options must have clear and explicit meanings consistent with the purpose of each item. Further, the response options should offer a clear distinction between choices, represent similar intervals and the number of options should be justified empirically [32]. The problems associated with raw rating scales and response options need to be treated with rigour in the construction and application, and it has been argued for the use of methodologies such as Rasch measurement [33]. In this context, our testing of response categories demonstrated that the four response options in the VQ6 questionnaire are both solid and justified from an empirical standpoint.

The Rasch analysis further confirmed the validity and reliability of the VQ6, demonstrating a high precision, item fit and targeting in the population of patients with PAD, and comparable to previously reported results by Nordanstig et al. [19]. The person separation and person separation reliability index (PSI) were, however, somewhat higher than in the previous study [19], indicating a higher measurement precision (i.e, how many levels of person-ability that the measurement can discriminate). PSI indicates the extent to which scores are associated with random error and meet the definition as a reliability indicator. In previous studies, the internal consistency and PSI have showed nearly identical values [20]. There are several differences between PSI and Cronbach’s alpha. For example, PSI can be computed when data are incomplete, while complete data are required for alpha calculations. In the Rasch analysis, missing items affect the standard error of person location, not the ability to make an estimation. However, PSI will then decrease [25]. Nevertheless, no missing data was reported in the present study; and the PSI and alpha values were principally identical, indicating a good reliability.

The Rasch analysis of VQ6 showed a raw variance explained by measures of 72.0% compared to 62.7% in the study by Nordanstig et al. [19], implying that the items in the VQ6 measure the same underlying trait and no other dimensions, so-called unidimensionality. Thus, the six items that reflect the five dimensions of activity, pain, symptom, emotional and social aspects also manage to capture the latent trait of how PAD influences daily life. This is important since indistinctness whether two individuals with the same score can be considered as comparable may have consequences in therapeutic clinical trials for the selection for interventions for individual patients [34]. Finally, the VQ6 capacity in targeting was very good, and the level of disability reflected by the items and the ability of the respondents have about the same mean. There should be items reflecting the ability for poorly performing patients and items reflecting disability for the able patients [20]. This means that in this study there was little better capacity among the patients than the items were able to reflect.

The percentage agreement (PA) was somewhat poor in all six items in the VQ6, and no item exceeded 70%, which was the limit for acceptable agreement. However, a more recent study have defined poor PA to be less than 60% [35] (Bernhardsson & Larsson 2013) and only two items of VQ6 (item 1 and 4) were below this limit. Besides the use of different definitions for PA, another argument for interpreting PA with caution is that it does not correct for agreements that would be expected by chance [36]). Moreover, no systematic disagreement in either relative position (RP) or relative concentration (RC) could explain the PA. The relative rank variance (RV) was >0.1 in two of the VQ6 items (item 1 and 3 regarding activity), suggesting that the group of patients could be considered as heterogeneous. This could also mean that these items are sensitive to disturbing factors of the test situation [37]. The low percentage agreement for the other four items could not be explained by a systematic change either for the group or for the individuals. In test-retest analysis, it is important to select patients who have a stable condition [28]. It has previously been described that PAD could be a rather unstable condition, with good and bad days when recovering from a vascular treatment [38]. Thus, one month after treatment may be a somewhat too short time period for some patients to reach a stable condition. In theory, an early failure of the invasive vascular procedure could also markedly have influenced the stability of the condition in some of the tested patients. Furthermore, according to the cognitive interviews, the two-week recall period was considered as too short for reflecting the PAD course. This may explain the individual response variations observed in the present study. Yet, given that the VQ 6 sum score at the first and second assessment were highly correlated (*r* = 0.76), and that an acceptable ICC was observed (0.86), the test-retest properties of the VQ-6 overall could be considered acceptable.

### Limitations

The patients who answered the VQ6 twice were doing so for the test-retest purpose, and the responsiveness of the VQ6 was never tested. The responsiveness of an instrument is one important property, and this needs to be further validated in the target population. The majority of the patients had critical limb ischemia, which means that the result may be less representative for patients with claudication. Further, a few patients with popliteal aneurysm were included who do not actually reflect the intended target population for the VQ6. A critical aspect in test-retest studies is to select an appropriate time interval [28]. There is yet no evidence to guide the selection of the time interval when investigating test-retest reliability for health status measurements [39]. However, a general recommendation is intervals ranging from 2 to 14 days [40]. The patients in the present study were all included at their one-month follow-up visit after an invasive vascular treatment, and they were instructed to fill out the second questionnaire at home one week after their visit at the open clinic and to return it in the prepaid envelope. This means that we do not know exactly when they answered the second questionnaire and there may have been some individual variation in timing of the second assessment. The effect of memory is another critical point in test-retest studies. The VQ6 is a short instrument, and it may be easy to remember the six items. However, the variation in answers between time points indicates that this risk has been minimised. Some of the patients (10%) who got a second questionnaire did not return the questionnaire. The reason for this is unknown, but it may depend on difficulties comprehending the items in VQ6 or just a lack of commitment. However, the results from the cognitive interviews suggest that the six items were both relevant and understandable, which is why the former reason seems unlikely.

## Conclusion

The VQ6 has acceptable to good psychometric properties with regard to data quality, scale assumptions, targeting, validity and reliability. Further, the VQ6 is easy to use and comprehend by the target PAD population. The ideal instrument for measuring health-related quality of life should be brief, easy to use, accurate and effective for determining HRQoL in patients with PAD, and it should be useful in both clinical and research settings. The VQ6 seems to fulfil these criteria, and it could be a valuable tool to enhance understanding of HRQoL in patients with PAD and to guide and to improve treatment strategies in this patient population.

## Abbreviations

ANOVA: One-way analysis of variance
CI: Confidence Interval
DIF: Differential item functioning
EQ5D: EuroQol 5 dimensions
HRQoL: Health-related quality of life
ICC: Intra class correlation coefficient
MCS: Mental component summary score
PA: Percentage agreement
PAD: Peripheral arterial disease
PCS: Physical component summary score
PSI: Person separation index
RC: Relative concentration
RP: Relative position
RV: Relative rank variance
SF-36: Short Form health survey 36
VascuQoL: Vascular Quality of Life Questionnaire
VQ6: VascuQoL-6
